# Pragmatic assessment of exercise in routine care using an MDHAQ: associations with changes in RAPID3 and other clinical variables

**DOI:** 10.1186/s13075-016-1095-x

**Published:** 2016-09-06

**Authors:** Isabel Castrejón, Yusuf Yazici, Selda Celik, Theodore Pincus

**Affiliations:** 1 Department of Medicine, Division of Rheumatology, Rush University Medical Center, 1611 West Harrison Street, Suite 510, Chicago, IL 60612 USA; 2 Department of Medicine, Division of Rheumatology, New York University School of Medicine and NYU Hospital for Joint Diseases, New York, NY USA

**Keywords:** Patient self-report, Exercise, Disease activity, MDHAQ, RAPID3, Quantitative assessment

## Abstract

**Background:**

Exercise is associated with major benefits in patients with rheumatic diseases for both cardiovascular and rheumatic status. However, information about exercise generally is not collected systematically in routine rheumatology care. A multidimensional health assessment questionnaire (MDHAQ), which was designed for busy clinical settings, includes a query about exercise status. We analyzed possible associations between change in MDHAQ exercise scores and other MDHAQ measures in patients with various rheumatic diseases over one year.

**Methods:**

In one rheumatology clinical setting, all patients, regardless of diagnosis, complete an MDHAQ before seeing a rheumatologist. The MDHAQ includes scores for physical function, pain, and patient global estimate, compiled into an index, routine assessment of patient index data (RAPID3), as well as a self-report joint count and a query about exercise. Patients were classified into four groups according to their exercise status at baseline and one year later as: EXER-Yes (regular exercise), EXER-Yes; EXER-No (no regular exercise), EXER-Yes; EXER-Yes, EXER-No; and EXER-No, EXER-No. These groups were compared using the chi square and Kruskal-Wallis tests and analysis of variance (ANOVA).

**Results:**

Patients who reported regular exercise at baseline were younger, had higher formal education, and better clinical status than other patients. The EXER-No, EXER-Yes group had greater improvement in other MDHAQ variables than patients in the other three groups. By contrast, the EXER-Yes, EXER-No group was the only group with poorer status one year later.

**Conclusions:**

The MDHAQ exercise query indicates that regular exercise is associated with better clinical status. Patients in the EXER-No, EXER-Yes group reported the best clinical improvement, although it is not known whether exercise preceded or followed the improved clinical status.

## Background

The traditional approach to treatment of rheumatoid arthritis (RA) emphasized rest, and regular exercise was discouraged [1, 2]. However, over the last two decades exercise has been recognized as beneficial to people with RA and other rheumatic diseases, not only for cardiovascular and general fitness, but also for better rheumatologic clinical status [3–5]. Furthermore, the absence of exercise carries a similar risk for mortality 5 years later as﻿ the risk associated with smoking in a general elderly, non-diseased population [6].

Despite extensive evidence on the value of exercise in RA, most patients seen in rheumatology care do not participate in regular exercise. For example, in the QUEST RA database of 21 countries, regular exercise was reported by only 29 % of 5235 patients with RA, including more than 50 % in only 2 countries (Finland and Hungary), 21–50 % in 12 countries (Ireland, The Netherlands, Sweden, Lithuania, Greece, Denmark, USA, Germany, Canada, Estonia, and Serbia) and fewer than 20 % in 7 countries (Spain, Latvia, Poland, Turkey, France, Italy, and Argentina) [7].

The safety and effectiveness of exercise programs have been established primarily in structured research studies rather than in routine rheumatology care [3, 8–10]. In most busy clinical settings, data on exercise in individual patients generally are not collected systematically, if at all, and few data are available on levels of exercise. The capacity to collect information from patients on exercise in a pragmatic, feasible manner, without additional effort on the part of a rheumatologist, could advance the care and outcomes of patients with﻿ RA and other rheumatic diseases.

A multidimensional health assessment questionnaire (MDHAQ) is designed to be completed by patients in routine care [11]. The MDHAQ includes scores for physical function, pain and patient global estimate of status (PATGL) to provide a composite routine assessment of patient index data score of 0–30 (RAPID3) [12], as well as a query about exercise status.

Our objective was to analyze possible associations of demographic and clinical status variables with patient self-report exercise status at baseline and one year later in unselected patients with different rheumatic diseases seen in routine care.

## Methods

### Patients

Since July 2005, all patients seen at the Seligman Center for Advanced Therapeutics at the NYU Hospital for Joint Diseases complete an MDHAQ at all visits, while waiting to see the rheumatologist, in the infrastructure of routine care. MDHAQ scores, laboratory tests, and medications are recorded in a database. A physician global estimate of status (DOCGL) also is collected in routine care. A primary diagnosis was assigned by the treating physician according to ICD-9 codes. Patients with available data on exercise status at baseline and one year later through April 2011 were included in the study. The Institutional Review Board (IRB) of New York University School of Medicine approved a retrospective chart review of the data; therefore, no specific consent was needed for this study.

### Patient self-report MDHAQ

The MDHAQ is a two-page patient self-report questionnaire [13–15] developed for quantitative assessment in routine clinical care [16], which includes physical function in 10 activities, scored 0–3 (0 = without any difficulty, 1 = with some difficulty, 2 = with much difficulty, and 3 = unable to do) for a total physical function (FN) score of 0–30. The raw FN 0–30 score for the 10 activities is converted to 0–10 using a template on the MDHAQ, and added to two 0–10 visual analog scale (VAS) scores for pain and patient global estimate of status (PATGL), to provide a composite 0–30 RAPID3 score [12].

The MDHAQ also includes an RA disease activity index (RADAI) self-report joint count [17], which queries patients to score pain in 16 specific joint groups, 8 each on the right and left sides: fingers, wrists, elbows, shoulders, hips, knees, ankles, and toes. Scoring options are: 0 = none, 1 = mild, 2 = moderate, or 3 = severe pain; the total score range is 0–48 [17]. Queries about the back and neck in an identical format are added on the MDHAQ, but not included in scoring, to be comparable to RADAI scores elsewhere.

The MDHAQ also includes scores for anxiety, depression, and sleep quality, a 60-item symptom checklist, review of 12 recent medical history events, morning stiffness, fatigue VAS, change in status, demographic data, and a query about exercise [12, 13].

### Self-report exercise status

The MDHAQ exercise query is: “How often do you exercise aerobically (sweating, increased heart rate, shortness of breath) for at least one-half hour?” Five possible response options are: “3 or more times a week”, “1–2 times per week”, “1–2 times per month”, “do not exercise regularly”, “cannot exercise due to disability/handicap”. In this study, no patient selected the option “1–2 times per month” at baseline or a year later. Responses of weekly exercise ≥3 times and 1–2 times were pooled as “regular exercise” (EXER-Yes). Responses of “do not exercise regularly” or “cannot exercise due to disability/handicap” were pooled as “no exercise” (EXER-No) [7]. Patients were classified into four groups according to exercise status at baseline and one year later: exercise at both baseline and one year later (EXER-Yes, EXER-Yes), no exercise at baseline but exercise one year later (EXER-No, EXER-Yes), exercise at baseline but not one year later (EXER-Yes, EXER-No), and no exercise at both baseline or one year later (EXER-No, EXER-No).

### Statistical analysis

The MDHAQ is incorporated into the medical record for clinical care. The data also are entered into a database, which includes demographic, MDHAQ, medication and laboratory data. Means and standard deviations (SD) were calculated for normally distributed data, and medians and interquartile ranges (IQR) for non-normally distributed data. Baseline differences between patients in the four groups according to exercise status at baseline and one year later were compared using the chi square and Kruskall-Wallis tests. Mean change from baseline to one-year follow up was compared by analysis of variance (ANOVA) and presented as percentage of change. A negative change indicates improvement and a positive change indicates worsening. Bivariate regression analyses were performed to identify baseline variables associated with exercise or no exercise; variables that were significant in bivariate analyses were included as covariates in multivariate logistic regression analyses. All analyses were performed using STATA 12.0® for Mac (StataCorp LP, College Station, TX, USA).

## Results

### Patients

Overall, 795 patients with various rheumatic diagnoses were included in this study. The more prevalent diagnoses were RA (221 patients (27.8 %)), systemic lupus erythematosus (125 patients (15.7 %)), psoriatic arthritis (86 patients (10.8 %)), and osteoarthritis (58 patients (7.3 %)); 305 patients (38.4 %) had other diagnoses. Mean age was 51.1 years, 74.9 % were women, and 59 % were Caucasian. The proportion of patients with >12 years of education was 93 %, substantially higher than in the general population and in most patients seen in rheumatology clinical settings [18–20].

### Exercise performance and clinical status at baseline and one year later

Participation in exercise at baseline and one year later (EXER-Yes, EXER-Yes) was reported by 324 patients (40.2 %), compared to 126 (15.8 %) who reported no exercise at baseline and exercise one year later (EXER-No, EXER-Yes), 77 (9.7 %) who reported exercise at baseline and no exercise at one year (EXER-Yes, EXER-No), and 268 (33.7 %), who reported no exercise at both time points (EXER-No, EXER-No) (Table 1). Patients who reported exercise at baseline and one year later (EXER-Yes, EXER-Yes group) had the lowest MDHAQ and DOCGL scores, indicating the best clinical status (*p* < 0.001) (Table 1). By contrast, patients in the EXER-No, EXER-No group were older, had a lower formal educational level, were more likely Hispanic, and had the poorest MDHAQ scores for physical function, pain, PATGL, RAPID3, fatigue, RADAI, and DOCGL compared to patients in the other groups (*p* < 0.001) (Table 1).

**Table 1:** Baseline demographic and clinical characteristics of patients according to self-reported exercise status at baseline, grouped by status at baseline and one year later

	All patients *N* = 795	Groups of patients by exercise status at baseline and one year later				
		EXER-Yes, EXER-Yes *N* = 324 (40.7 %)	EXER-No, EXER- Yes *N* = 126 (15.8 %)	EXER-Yes, EXER- No *N* = 77 (9.7 %)	EXER-No, EXER-No *N* = 268 (33.8 %)	*P*
Demographic variables						
Age, years, mean (SD)	51.1 (16)	43.7 (15.0)	45.3 (16.6)	48.3 (15.0)	51.2 (15.9)	<0.001
Gender, % female	74.9 %	73.3 %	72.9 %	76.4 %	77.2 %	0.68
Ethnicity						0.003
Percent (%) Caucasian/ Black/ Hispanic/ Asian/﻿﻿ Other	59 %/ 12 %/ 17 %/ 8 %/﻿ 4 %	62 %/ 12 %/ 13 %/ 10 %/ 3 %	55 %/ 18 %/ 14 %/﻿ 4 %/ 9 %	58 %/ 7 %/ 19 %/ 13 %/ 3 %	56 %/ 10 %/ 24 %/﻿ 7 %/ 3 %	
Formal educational level, years	16 (13–18)	16 (15–18)	16 (14–16)	16 (13.5–18)	15 (12–17)	<0.001
MDHAQ variables						
Physical function (0–10)	1.3 (0.3–3.3)	0.7 (0–2.3)	1.7 (0.3–3.7)	2 (0.3–3)	2.3 (0.7–4)	<0.001
Pain VAS (0–10)	5 (2–7.5)	3.5 (1–6)	5.5 (2.5–7)	4 (2–6.5)	6 (3.5–8)	<0.001
Patient global estimate VAS (0–10)	5 (2–6.5)	3 (1–5.5)	4.5 (2–7)	4.5 (2–6)	5.5 (4–7.5)	<0.001
RAPID3 (0–30)	11.2 (5–16.5)	8.2 (3.5–13.3)	11.8 (5.7–18)	10.3 (4.7–15.5)	14 (8.5–19)	<0.001
Fatigue VAS (0–10)	4.5 (1–7.5)	3 (1–7)	4.5 (0.5–7)	5.5 (2–7)	5.5 (2.5–8)	<0.001
RADAI (0–48)	6 (2–14)	4 (1–10)	8 (3–15)	9 (2–17)	9 (3–19)	<0.001
Physician global estimate VAS (0–10)	3 (2.5–3.5)	2.5 (2–3.5)	3 (2.5–4.5)	3 (3–3.5)	3 (2.5–4)	<0.001

Values are median and interquartile range unless otherwise indicated. *P* values were obtained using Kruskall-Wallis one-way analysis of variance (ANOVA) for continuous variables with no normal distribution, ANOVA for variables with normal distribution, and the chi square test for categorical variables. *Exer* exercise, *MDHAQ* multidimensional health assessment questionnaire *RAPID3* routine assessment of patient index data3, *RADAI* rheumatoid arthritis disease activity index, *VAS* visual analog scale

### Associations between demographic and clinical variables and changes in exercise status from baseline to one-year follow up

Patients in the entire cohort improved (negative change) in each MDHAQ variable, including 15.5 % improvement in RAPID3 and 26.7 % improvement in RADAI scores. The highest level of improvement in RAPID3 and other MDHAQ scores (23.2–40 %) was in patients who reported no exercise at baseline but did report exercise one year later (EXER-No, EXER-Yes) (Table 2). By contrast, the patient group that reported exercise at baseline but not at one year later (EXER-Yes, EXER-No) was the only group with unchanged or poorer status one year later, albeit less than 6 % poorer, except for 15 % poorer physical function. The percentage improvement according to DOCGL did not differ significantly in the four groups (Table 2).

**Table 2:** Change from baseline to one-year follow up

	All patients *N* = 795	Mean (%) change between baseline and one year according to self-reported exercise status				*P* ANOVA
		EXER-Yes, EXER-Yes *N* = 324 (40.7 %)	EXER-No, EXER-Yes *N* = 126 (15.8 %)	EXER-Yes, EXER-No *N* = 77 (9.7 %)	EXER-No, EXER-No *N* = 268 (33.7 %)	
Physical function (0–10)	-0.19 (-14.6 %)	-0.2 (-13.3 %)	-0.8 (-34.7 %)	0.3 (15.0 %)	-0.01 (-0.4 %)	<0.001
Pain VAS (0–10)	-0.77 (-15.4 %)	-0.5 (-12.8 %)	-1.7 (-34.0 %)	-0.2 (-4.5 %)	-0.8 (-14.3 %)	<0.001
Patient global estimate VAS (0–10)	-0.71 (-14.2 %)	-0.4 (-11.1 %)	-1.8 (-40.0 %)	0.2 (4.8 %)	-0.8 (-14.8 %)	<0.001
RAPID3 (0–30)	-1.74 (-15.5 %)	-1.2 (-13.3 %)	-4.4 (-37.3 %)	0.3 (2.8 %)	-1.7 (-12.4 %)	<0.001
Fatigue VAS (0–10)	-0.50 (-11.1 %)	-0.3 (-7.9 %)	-1.0 (-23.2 %)	-0.3 (-6.2 %)	-0.6 (-11.3 %)	0.13
RADAI (0–48)	-1.6 (-26.7 %)	-2.1 (-28.0 %)	-3.9 (-37.9 %)	0.1 (1.2 %)	-0.3 (-2.4 %)	0.004
Physician global estimate VAS (0–10)	-1.1 (-36.7 %)	-0.9 (-30 %)	-1.4 (-40.0 %)	-1.5 (-48.4 %)	-1.1 (-35.5 %)	0.34

Negative change indicates improvement and positive change worsening. Mean (%) change between baseline and one year. *ANOVA* analysis of variance, *EXER* exercise, *RAPID3* routine assessment of patient index data3, *RADAI* rheumatoid arthritis disease activity index, *VAS* visual analog scale

In bivariate logistic regression including all patients, older age (>50 years), lower educational level, poor physical function (score >1.3), high pain (score >5), high baseline RAPID3 (score >6), and high fatigue (score >5) at baseline were associated significantly with no exercise at baseline (Table 3). In multivariate analyses, older age, lower educational level, poorer function, and high fatigue remained significant independently; baseline age over 50 years and poor physical function were most strongly associated with no exercise.

**Table 3:** Association between physical inactivity and demographic and clinical variables at baseline according to the MDHAQ

	Bivariate logistic regression model		Multivariate logistic regression model	
	OR (95 % CI)	*P*	OR (95 % CI)	*P*
Demographic variables				
Age (>50 years old)	2.39 (1.71–3.34)	<0.001	1.94 (1.30–2.89)	0.001
Female	1.23 (0.84–1.81)	0.28	---	---
Education	0.87 (0.82–0.92)	<0.001	0.89 (0.84–0.95)	0.001
Ethnicity (Caucasian)	0.77 (0.54–1.10)	0.15	---	---
MDHAQ variables				
Physical function (>1.3)	3.01 (2.14–4.23)	<0.001	1.89 (1.13–3.18)	0.01
Pain (>5)	2.56 (1.82–3.59)	<0.001	1.46 (0.88–2.42)	0.14
RAPID3 (>6)	2.61 (1.76–3.85)	<0.001	0.82 (0.44–1.51)	0.52
Depression (≥1)	1.29 (0.87–1.92)	0.20	---	---
Fatigue (>5)	2.44 (1.74–3.42)	<0.001	1.84 (1.16–2.92)	0.009

*MDHAQ* multidimensional health assessment questionnaire, *OR* odd ratio, *CI* confidence interval, *RAPID3* routine assessment of patient index data3

## Discussion

Our results indicate that 50.4 % of patients seen in routine care at one site in New York reported exercising one or more times per week at baseline [7]. This proportion was higher than seen in 31 % of US patients in the QUEST-RA database (29.1 % of 5235 patients from 21 countries) in response to the same query [7]. This finding may be explained in part by the considerably higher educational level of patients seen at NYU, with a median level of 16 years of formal education of, compared to 13.5 years for US patients in QUEST-RA and 10.9 years in all patients, as more educated patients are significantly more likely to exercise regularly [7]. 

Baseline MDHAQ scores were lowest (indicating better status) in patients who reported exercise at both time points, and highest in patients who reported no exercise at either time point. Mean changes in each MDHAQ score were highest in patients who reported no exercise at baseline, but regular exercise one year later, consistent with an observation that the greatest benefit of exercise is seen among the least fit individuals [21]. No significant differences were seen in the percentage of improvement according to DOCGL in any group. However, there was numerically greater improvement in the group which reported exercise at baseline but no exercise at one year, consistent with evidence of discordance between PATGL and DOCGL reported in an earlier study of many patients in the same cohort as in this report [22].

This study has several important limitations. The primary limitation involves the absence of information on causality - whether a change in exercise status may have preceded or resulted from a change in clinical status. Other limitations include the absence of any specific information on the type of exercise performed, whether rheumatologists reviewed the information about exercise, or how they might have used this information to counsel patients. Nonetheless, the data indicate that a simple query on an MDHAQ can assess exercise status quantitatively in routine care with no extra effort on the part of the rheumatologist or office staff﻿,﻿ and indicates changes associated with other variables. Long-term exercise appeared not only to have no deleterious effects, but on the contrary, clinical benefit was seen, confirming results from previous studies [3–5].

## Conclusions

In summary, our data indicate the pragmatic feasibility of screening for patient exercise status as a simple procedure in routine care, with no extra work on the part of the doctor, by having the patient complete an MDHAQ in the waiting area prior to seeing the rheumatologist. Such assessment provides a relatively easy strategy for rheumatologists to encourage patients to exercise regularly. Furthermore, eliciting the data through self-report could stimulate patients concerning the desirability of exercise in rheumatic conditions, and possibly motivate behavior change over time. The data indicate that the differences in the self-report exercise query were correlated with changes in clinical status, as no exercise at baseline but regular exercise one year later was associated with significant clinical improvement, although they do not explain whether improved clinical status resulted from or preceded regular exercise. Initial assessment of exercise in a feasible manner on a patient self-report MDHAQ appears to be a potentially helpful measure toward encouraging exercise as a component of care for all rheumatic diseases, toward better patient care and long-term outcomes, with no additional effort on the part of the rheumatologist.

## Abbreviations

ANOVA: analysis of variance
DOCGL: physician global estimate of status
EXER-No: no regular exercise
EXER-Yes: regular exercise
FN: physical function
IQR: interquartile range
MDHAQ: multidimensional health assessment questionnaire
NYU: New York University
PATGL: patient global estimate of status
RA: rheumatoid arthritis
RADAI: rheumatoid arthritis disease activity index
RAPID3: routine assessment of patient index data
SD: standard deviation
VAS: visual analog scale
